# A Dual-modality Smartphone Microendoscope for Quantifying the Physiological and Morphological Properties of Epithelial Tissues

**DOI:** 10.1038/s41598-019-52327-x

**Published:** 2019-10-31

**Authors:** Xiangqian Hong, Tongtong Lu, Liam Fruzyna, Bing Yu

**Affiliations:** Department of Biomedical Engineering, Marquette University and Medical College of Wisconsin, Milwaukee, WI 53233 USA

**Keywords:** Biomedical engineering, Imaging and sensing, Optical spectroscopy

## Abstract

We report a nonconcurrent dual-modality fiber-optic microendoscope (named SmartME) that integrates quantitative diffuse reflectance spectroscopy (DRS) and high-resolution fluorescence imaging (FLI) into a smartphone platform. The FLI module has a spatial resolution of ~3.5 µm, which allows the determination of the nuclear-cytoplasmic ratio (N/C) of epithelial tissues. The DRS has a spectral resolution of ~2 nm and can measure the total hemoglobin concentration (THC) and scattering properties of epithelial tissues with mean errors of 4.7% and 6.9%, respectively, which are comparable to the errors achieved with a benchtop spectrometer. Our preliminary *in vivo* studies from a single healthy human subject demonstrate that the SmartME can noninvasively quantify the tissue parameters of normal human oral mucosa tissues, including labial mucosa tissue, gingival tissue, and tongue dorsum tissue. The THCs of the three oral mucosa tissues are significantly different from each other (p ≤ 0.003). The reduced scattering coefficients of the gingival and labial tissues are significantly different from those of the tongue dorsum tissue (p < 0.001) but are not significantly different from each other. The N/Cs for all three tissue types are similar. The SmartME has great potential to be used as a portable, cost-effective, and globally connected tool to quantify the THC and scattering properties of tissues *in vivo*.

## Introduction

The cancer burden increased to 18.1 million new cases and 9.6 million cancer deaths worldwide in 2018^1^. Both the incidence and mortality rates of cancer are disproportionately high in low- and middle-income countries (LMICs)^1^. Malignancies of epithelial tissues account for 80% to 90% of all cancer cases^2^. Intraepithelial neoplasia (IEN) is a precancerous condition associated with an increased risk of developing into cancer. Some of the most common IENs include cervical intraepithelial neoplasia (CIN), gastrointestinal intraepithelial neoplasia (GIN), and oral intraepithelial neoplasia (OIN). Neoplastic epithelial tissue exhibits significant changes in its physiological and morphological characteristics such as angiogenesis, a degradation in extracellular collagen networks, and increased nuclear size and DNA content^3^. An accurate characterization of these changes in an early stage is the key to the prevention of cancer because it greatly increases the chance for a successful treatment if the changes are malignant and reduces the cost for unnecessary diagnoses and treatments (e.g., biopsies and follow-ups) if the changes are benign. However, due to poor medical conditions and a lack of resources, the benefits of early prevention and diagnosis methods such as human papillomavirus (HPV) vaccination or regular screening have yet to be realized in LMICs. There is an urgent need for a more affordable, portable and simple technique to accurately measure the physiological and morphological properties of tissues *in vivo* in LMICs.

Optical imaging and spectroscopy are powerful tools for the quantitative characterization of physiological and morphological changes in neoplastic epithelial tissues. Various optical techniques, including confocal microscopy^4,5^, narrowband imaging (NBI)^6,7^, optical coherent tomography (OCT)^8,9^, photoacoustic imaging (PAI)^10,11^, diffuse reflectance spectroscopy^12–14^, near-infrared Raman spectroscopy^15,16^, and fluorescence imaging^17–19^ have shown potential in improving precancer detection. The major drawbacks of these optical techniques are the use of sophisticated and expensive components, making them unaffordable in LMICs. There has been increasing interest in the past decade in converting mobile phones to portable and cost-effective optical devices for medical applications. For instance, Breslauer *et al*.^20^ reported a cellphone-mounted microscope for the screening of hematologic diseases. Red blood cells were visualized in both brightfield and fluorescence with a resolution of 1.2 µm. Switz *et al*.^21^ transformed a mobile phone to a microscope by attaching a reversed camera lens to the phone camera. This design enlarged the field of view of the microscope and allowed high-quality imaging of red and white blood cells. Tseng *et al*.^22^ reported a lightweight and lens-free imaging attachment that can convert a mobile phone into a holographic microscope. Smartphone-based endoscopes for various applications were also reported by different groups. Wu *et al*.^23^ recently demonstrated a smartphone-based otorhinoendoscope for the diagnosis of ear and nose diseases. The diagnosis results based on measurements taken from six patients using the smartphone-based device were comparable to those of a traditional otorhinoendoscope. Similarly, Endoscope-i Ltd. commercialized a smartphone-based otoendoscope system developed by Jongsma *et al*. in 2012^24^. Smartphone spectrometers with nanometer to subnanometer accuracy have also been reported. For example, Gallegos *et al*.^25^ demonstrated the use of a smartphone spectrometer to measure shifts in the resonance wavelength of a label-free photonic crystal biosensor with an accuracy of 0.009 nm. Wang *et al*.^26^ proposed multiple methods for the implementation of smartphone-based spectroscopy. Smith *et al*.^27^ developed two attachments that transform a phone camera into either a microscope or a spectrometer.

While single-smartphone imaging and spectroscopy methods are being actively studied, few studies have been performed on dual- or multimodality smartphone-based endoscopy, which has the potential to provide the physician with complementary information about a lesion in question with improved sensitivity and specificity. A handheld colposcopy system developed by MobileODT can simultaneously perform bright-field, polarization-difference, and spectral imaging with applications for cervical cancer detection in developing countries^28^. While the MobileODT imaging system is a powerful tool for cervical cancer imaging, the device uses a rigid imaging tube that contains sophisticated imaging optics and requires a dedicated engineer to operate the instrument and an experienced physician to read the images; thus, its application in LMICs is limited.

We previously reported a visible to near-infrared G-Fresnel spectrometer for the measurement of hemoglobin content in tissue-mimicking phantoms^29^ and a smartphone-based fluorescence microendoscope using a fiber-optic imaging bundle for subcellular resolution imaging^30^. The previous design of the G-Fresnel spectrometer included an external CMOS image sensor, a customized G-Fresnel grating (600 lines/mm) and a 25 µm slit. The G-Fresnel grating acts as a transmission grating and a focusing lens. The G-Fresnel spectrometer is connected to a smartphone using a micro-USB cable. When used with an external 20 W tungsten halogen lamp and six 400 µm fibers for DRS, the system has an optical resolution of ~5 nm and a typical integration time of 3.6 seconds. While the G-Fresnel spectrometer is very compact in size, its poor spectral resolution, long integration time, and requirement for a high-power large tungsten halogen lamp make it difficult to integrate the spectrometer with an FLI device into a very portable and battery-powered device. In addition, the smartphone for the G-Fresnel spectrometer cannot be charged during the measurement due to the use of the micro-USB port for image collection. More importantly, both devices are standalone instruments in which data and images are postprocessed manually on a computer; thus, the measurement results are not immediately available on the device.

In this report, we describe a dual-modality fiber-optic microendoscope (named SmartME) with a very small form factor that integrates high-resolution FLI and quantitative DRS into a smartphone platform for the noninvasive quantification of the physiological and morphological properties of epithelial tissues. When used with the customized Android Application (App) and server software, the device can be used to create a smart, affordable and globally connected diagnostic solution: the SmartME and App collect and transmit diffuse reflectance spectra and high-resolution fluorescence images of epithelial tissue at the point of care, and the server with loaded spectrum and image processing software extracts the physiological and morphological parameters of the tissue and returns the results to the SmartME. While the FLI module is moderately improved over the previous version, the DRS module and the fiber-optic probe have been completely redesigned. First, the rear camera (with a lens kit) of the smartphone is used to collect the diffracted light instead of using an extra CMOS sensor. This design makes the micro-USB port available for charging and other uses. A white LED, 100 µm optical slit and a transmission grating are used to obtain a sufficient signal and an adequate resolving power. A comparison between the designs of the SmartME device and the G-Fresnel spectrometer is provided in the Supplementary information. With the new design, the spectral resolution is significantly improved (from 5 to 2 nm), and the integration time is reduced from 3.6 to 0.5 seconds with a 20 mW white LED as the light source and only three smaller (200 µm) illumination and detection fibers. The combined SmartME has been characterized in terms of spatial resolution using a 1951 USAF resolution test target and in terms of accuracy by measuring the optical properties of tissue using liquid tissue phantoms. Preliminary *in vivo* measurements from a single human subject have been taken on healthy human oral tissues, including labial mucosa tissue, gingival tissue and tongue dorsum tissue (the upper surface of the tongue), to demonstrate the capability of the SmartME in differentiating different types of normal epithelial tissues.

## Materials and Methods

### SmartME instrument

**Figure 1:**
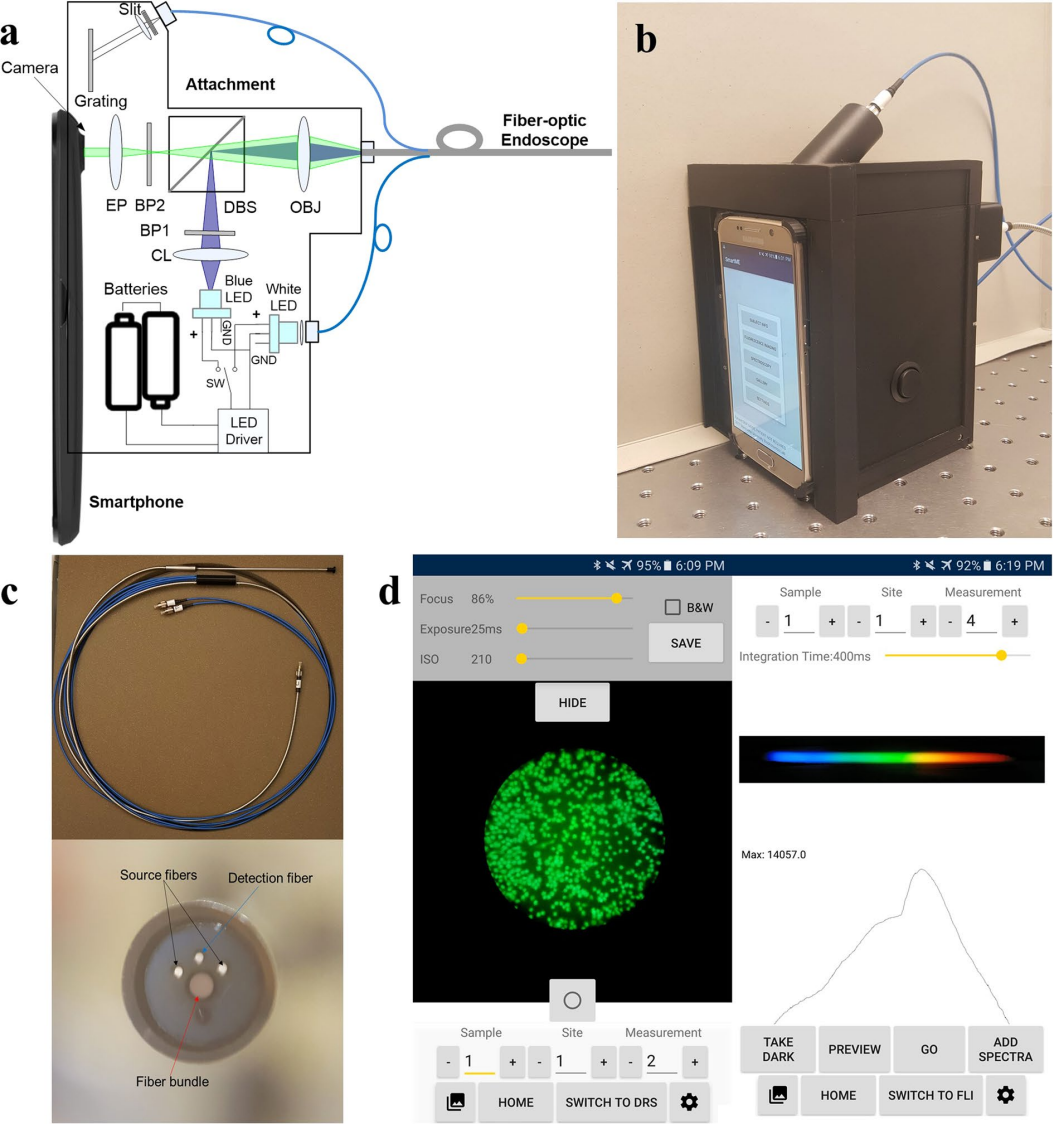
The SmartME system. (**a**) A schematic of the SmartME device; (**b**) a picture of the SmartME with the smartphone and fiber-optic endoscope attached; (**c**) a picture and distal-end view of the miniature dual-modality fiber-optic endoscope; and (**d**) screenshots of the SmartME App user interfaces in FLI (left) and DRS (right) modes.

A schematic diagram and picture of the SmartME device are shown in Fig. 1a,b, respectively. The system consists of a smartphone (Samsung Galaxy S6), a miniature fiber-optic endoscope, a phone attachment containing the imaging optics, and an App. The system includes two functional channels: an FLI channel and a DRS channel. The details of the FLI channel have been described elsewhere^30^. Briefly, a blue LED (455 nm, M455L3, Thorlabs) coupled with a condenser lens (CL, ACL2520U-A, Thorlabs) and a shortpass excitation filter (BP1, FF01-452/45, Semrock) are used for the fluorescence excitation. The excitation beam is redirected by a dichroic beamsplitter (DBS, AT485DC, Chroma Technology) towards a 10× microscope objective that focuses the excitation light onto the proximal end of a fiber bundle in the endoscope. The fluorescence emissions from a stained tissue sample collected by the same image bundle travel through the 10× objective (OBJ) lens, the DBS, an emission filter (BP2, FF01-550/88, Semrock) and a 16× eyepiece (EP) in sequence and then are captured by the smartphone camera sensor. The 600 μm image bundle (FIGH-30-650S, Fujikura) consists of ~30,000 individual fibers. The center-to-center distance between each fiber is ~3.3 μm, and a single fiber has a diameter of ~2 μm. The selection of the blue LED and filter set for the FLI is based on the use of proflavine, which has a preferential cell nucleus staining property, as the fluorescence dye. The absorption peak of proflavine in water at PH = 7 is approximately 445 nm, and the fluorescence emission is centered at 515 nm with a quantum yield of 0.34. Studies on the early detection of neoplasia in different types of epithelial tissues, including Barrett’s esophagus, the cervix and the oral cavity, using proflavine have been reported by Muldoon *et al*.^31^ and Quinn *et al*.^19^.

To reduce the overall size and increase the power efficiency of the DRS device, a 20 mW white LED (MCWHF2, Thorlabs) is used in the design as the light source of the DRS channel. The white light is delivered to the tissue through two 200/220 µm multimode fibers attached along the imaging fiber bundle, as shown in the end view of the endoscope in Fig. 1c. The diffuse reflectance is collected by a single detection fiber, narrowed down by a 100 µm slit (S100RD, Thorlabs) inside the attachment and then collimated by a collimating lens. The collimated light is diffracted by a transmission grating with 1200 grooves/mm (GT13-12, Thorlabs) and then imaged on the rear camera of the smartphone. The source-detector separation (center-to-center distance between the source fibers and the detection fiber) at the distal end of the endoscope is 0.75 mm. The blue and white LEDs are powered by the same rechargeable batteries and turned on/off sequentially using a 3-position switch. Both the FLI and DRS channels are designed and optimized using a combination of the sequential and nonsequential modes in OpticStudio (Zemax LLC).

An App has been developed to configure and control the SmartME, preprocess the fluorescence and DRS images, and wirelessly communicate with a server where the data processing is performed. The App allows a user to set and save the camera parameters, initiate a measurement, store the measured images, perform simple analysis such as a grayscale image conversion and wavelength calibration, send the raw data to the server, and receive and display the analyzed results. Figure 1d shows screenshots of the App user interfaces in the FLI and DRS modes. While the data processing was performed manually in the previous designs, in the SmartME solution, custom software named “SmartME Uploader” has been developed and installed on the server to ensure a smooth connection between the SmartME and the server. The images and spectra collected by the App are wirelessly transmitted to the server through remote access with an IP address. Once the connection is established, the data files are uploaded to the server in real time whenever a measurement occurs. As soon as the data arrives at the server, the data processing modules are automatically activated. The processed results are then saved on the server and sent back to the SmartME device for display. The whole process, from the data transmission to the display of the results, takes a few seconds.

### SmartME characterization and calibration

**Figure 2:**
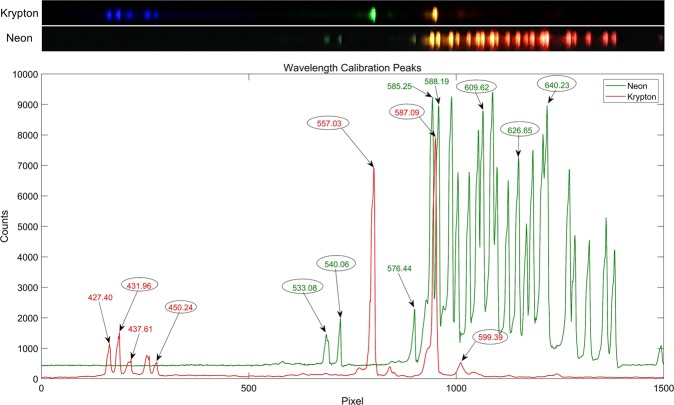
Cropped raw images (top) and spectra (bottom) of the calibration light sources captured by the SmartME DRS channel. The circled peaks were used for the wavelength calibration.

The spatial resolution of the smartphone FLI was estimated by imaging a 1951 USAF resolution test target. A green fluorescent reference slide (2273-G, Ted Pella Inc.) was placed under the resolution test target. The fiber-optic endoscope was brought in contact with the surface of the resolution target. The smallest pair of lines in the fluorescence images that could be successfully resolved was used to determine the spatial resolution of the SmartME. Neon and krypton calibration lamps were used together for a wavelength calibration of the DRS channel in the visible wavelength range from 430 nm to 640 nm. The raw DRS images and spectra of the calibration light sources captured by the SmartME are presented in Fig. 2. The wavelength calibration was performed by identifying the known peak wavelengths of the calibration lamps and the corresponding pixel positions of the peaks in the spectra. Ten peak wavelengths (circled in the figure) across the range of 430–640 nm were identified. The peak wavelengths and pixel positions were fitted by third-degree polynomial fitting to generate a calibration curve with a calibration error less than ±0.45 nm. The wavelength range of a spectral image of 1500 pixels in width was determined to be 395.5 nm to 693.3 nm. Therefore, the dispersion was approximately 0.2 nm/pixel. The full width at half maximum (FWHM) of monochromatic light is widely used to define the spectral resolution of a spectrometer. At the peak of 557.03 nm in Fig. 2, for example, the FWHM is 10 pixels. Therefore, the spectral resolution was estimated to be 2 nm (0.2 nm/pixel multiplied by 10), which was a significant improvement from the resolution of the previously reported G-Fresnel smartphone-based spectrometer. The calibration parameters were saved in the App so that the spectral image captured by the SmartME DRS channel can be automatically converted to a text file for display and data transfer.

To characterize the accuracy of the DRS channel in measuring the optical properties of epithelial tissues, 15 liquid phantoms with a mean (averaged over the wavelength range of 450–630 nm) absorption coefficient µ_a_(λ) between 0.4 cm^−1^–3 cm^−1^ and a mean reduced scattering coefficient µ_s_’(λ) between 7 cm^−1^–13 cm^−1^ were created using powdered human hemoglobin (H0267, Sigma Aldrich) as absorbers and 1.0-µm polystyrene microspheres (07310, Polyscience, Inc.) as scatterers. The expected µ_a_(λ) of the phantoms were independently determined from absorbance measurements of the stock hemoglobin solution using a UV/Vis spectrophotometer (LAMBDA 35, PerkinElmer Inc.) and scaled to the actual concentrations in the phantoms. The expected µ_s_’(λ) was computed from the density, size, and refractive index of the polystyrene spheres using Mie theory.

### *In vivo* study of healthy oral tissue

To test the feasibility of the SmartME for a quantitative characterization of the properties of epithelial tissue, three types of normal oral mucosa tissues, including labial mucosa tissue, gingival tissue and tongue dorsum tissue, from a single healthy human subject were imaged *in vivo*. The experiment was conducted on the researcher himself. The subject in this self-experiment fully understood the procedures and provided consent to participate in the experiment voluntarily. An exempt determination was received from Marquette University’s Institutional Review Board (IRB). All methods were performed in accordance with the relevant guidelines and regulations of the institution. Before the optical measurements, the volunteer was asked to rinse his mouth with a 0.9% saline solution. The SmartME was cleaned using 2% chlorhexidine digluconate in ethanol. Eight random sites were imaged from both labial mucosa and tongue dorsum tissues, while five random sites were measured from gingival tissues. All measurements were carried out in dim room light. As a dual-use instrument, the DRS measurements must be performed before the FLI measurements to avoid the influence of proflavine on the DRS readings. To take a measurement, the endoscope was held by the operator and slowly brought in gentle contact with the surface of the tissue. The diffuse reflectance spectra were collected by turning on the white LED and switching the SmartME App to DRS mode. Immediately following the DRS measurements, the endoscope was pulled away, and the surface of the measured area was topically stained using cotton swabs with 0.01% wt/vol proflavine for 15 seconds and then rinsed with PBS to remove the excessive dye. The fluorescence images were taken by positioning the endoscope at the same location, turning on the blue LED and switching the App to FLI mode. Each of the twenty-one tissue sites was imaged 5 times by slightly shifting the endoscope within the area of interest, resulting in a total of 105 diffuse reflectance spectra and 105 fluorescence images. A diffuse reflectance spectrum was also taken from a reflectance standard puck (Spectralon, Labsphere) for calibration purposes following the tissue measurements. A calibrated diffuse reflectance spectrum was obtained by dividing the tissue spectrum by the reference spectrum. All the calibrated spectral data and fluorescence images were saved in the App and wirelessly transferred to the remote server for further processing to extract the biological properties using the methods described below.

### Image processing and spectral analysis

While the fiber bundle introduces flexibility into the endoscopic imaging system, its honeycomb pattern artifact also introduces inherent artifacts into the images. A variety of methods for removing fiber bundle pixelation have been proposed. Generally, the processing methods can be classified into two groups: filtering and interpolation reconstruction. However, filtering blurs the image and reduces the contrast of the image. In this work, we employ the interpolation reconstruction method proposed by Elter *et al*.^32^ to eliminate the fiber pattern artifacts. First, the imaging area of the fiber bundle (600 µm in diameter) was cropped out of the raw image. The value of the center pixel within each fiber (which occupies multiple pixels in the smartphone camera) was extracted and assigned to the surrounding pixels within the fiber to reconstruct an image without the honeycomb pattern artifacts. The image was then converted to a binary image in ImageJ to calculate the N/C for the selected region of interest using the built-in particle analyzer. The N/C has been proven to be a useful parameter for characterizing epithelial neoplastic changes both *in vitro*^18^ and *in vivo*^19^.

A fast Monte Carlo (MC) inverse model of reflectance developed by Palmer *et al*.^33^ was employed on a remote server to analyze the calibrated diffuse reflectance spectra. The diffuse reflectance spectra of the liquid tissue phantoms were used to extract the values of µ_a_(λ) and µ_s_’(λ) between 450–630 nm. The inversion process was repeated 15 times; in each inversion, one phantom was selected as a reference to analyze all phantoms^33^. The percentage errors, which are the difference between the extracted and expected values of µ_a_(λ) and µ_s_’(λ) divided by the expected values, were computed. The reference phantom that generated the smallest errors was selected as a reference to invert the diffuse reflectance spectra of the oral tissues. The tissue hemoglobin concentrations were computed from the extracted µ_a_(λ) using the Beer-Lambert law.

## Results

### SmartME characterization and calibration

The actual size of the fluorescence image of the fiber bundle (0.6 mm in diameter) on the smartphone camera sensor is ~2.4 mm in diameter, resulting in a 4× magnification (2.4 mm/0.6 mm). In the fluorescence image (not shown) of the 1951 USAF resolution target, the intensity function across the black and white lines indicated that the difference in the intensities between the minimum (valley) and maximum (peak) of Element 2 in Group 7 was larger than 3 dB, while the intensity difference of Element 3 in Group 7 was less than 3 dB. Therefore, based on the FWHM, the SmartME could resolve the adjacent pair of lines in Element 2 in Group 7 of the resolution test target, which represented a resolution of ~3.5 μm. Recall that the center-to-center distance of the two adjacent fibers is approximately 3.3 μm, and the resolving power of the image bundle is very close to this limiting value. Our previous study^30^ showed that the FLI channel of the SmartME can image cell nuclei of monolayer cells *in vitro* and human oral mucosa *in vivo* when used with proflavine.

Average errors of 4.7% for µ_a_(λ) and 6.9% for µ_s_’(λ) were calculated across all 15 phantoms, which are comparable to the errors achieved with a benchtop spectrometer^34^. Phantom #9 yielded the smallest error and thus was selected as a reference to calibrate the SmartME DRS channel for the tissue studies.

### *In vivo* study of healthy oral tissue

**Figure 3:**
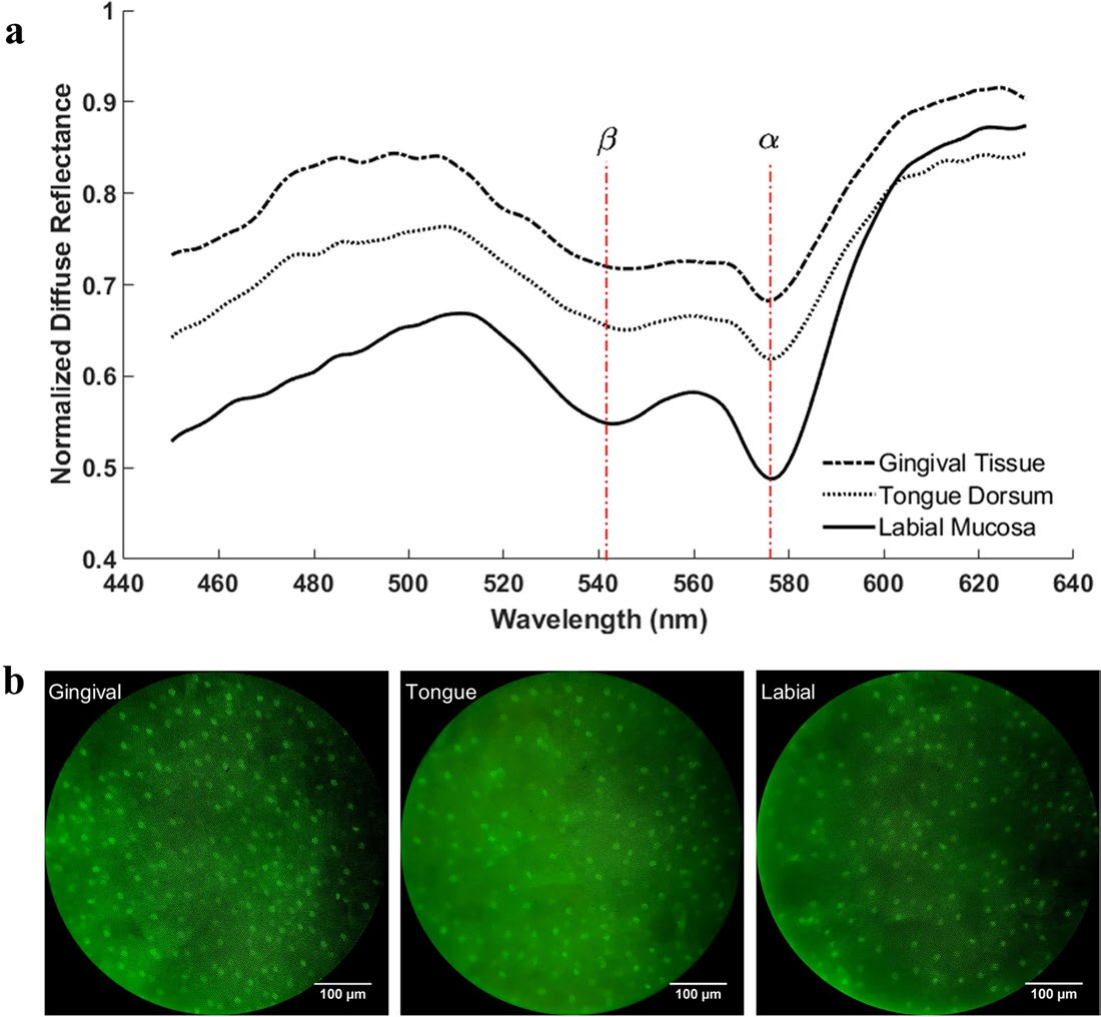
Measured tissue spectra and images from the normal gingival tissue, tongue dorsum tissue and labial mucosa tissue of a healthy subject *in vivo*. (**a**) Normalized representative diffuse reflectance spectra and (**b**) typical raw fluorescence images.

Figure 3a shows representative diffuse reflectance spectra from three normal oral sites that are normalized by (point-by-point) dividing by the puck spectrum. The two major absorption bands (α and β bands) of oxyhemoglobin are clearly visible in the spectra. The measured spectra of the three types of oral tissue are different from each other in shape and intensity, representing the difference in their underlying physiological and morphological characteristics. The typical raw fluorescence images taken from the three oral tissues are shown in Fig. 3b. Since proflavine effectively combines with DNA molecules, the bright spots that are clearly visible in the raw images are the cell nuclei.

**Figure 4:**
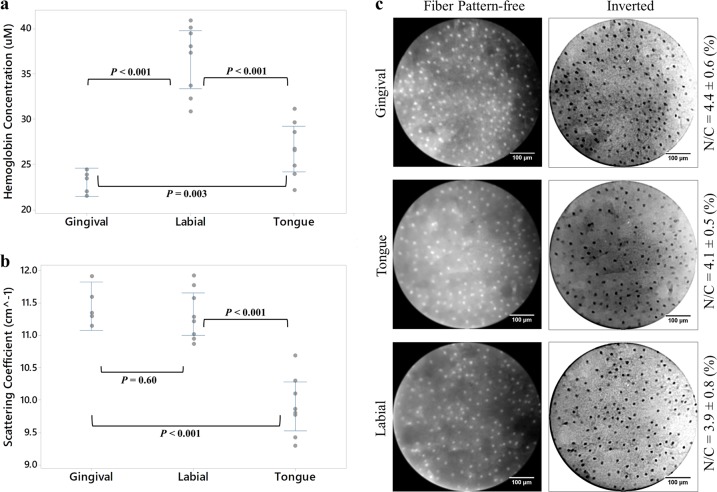
Interval plots of the (**a**) extracted total hemoglobin concentration and (**b**) extracted wavelength-averaged reduced scattering coefficient for three oral tissue types (gingival, labial and tongue). Each dot represents an average of 5 repeated measurements from the same tissue site. The p-values were calculated using a two-sided student t-test at a significance level of 5%. (**c**) Representative, fiber pattern-removed fluorescence images of the three oral tissues and the inverted images were used for the N/C calculation.

The reduced scattering coefficients µ_s_’(λ) and the total hemoglobin concentrations (THCs) were extracted from the diffuse reflectance spectra of the tissues using the MC inverse model. We assumed that both the µ_s_’(λ) and THCs are normally distributed, and student t-tests were performed using Minitab to test if there were significant differences among the three tissue types. Interval plots using the individual standard deviation of the extracted THCs and µ_s_’(λ) are presented in Fig. 4a,b, respectively. Each dot in the plots represents an average of 5 repeated measurements taken from the same tissue site.

Although the optical properties vary from site to site even for the same tissue type, the variation within each group of tissue type is generally smaller than that between different tissue types. The extracted THCs of the labial mucosa tissue are significantly higher than the THCs of the gingival and tongue tissues (p < 0.001). The THCs of the gingival and tongue tissues are also significantly different from each other (p < 0.003). While the extracted µ_s_’(λ) of the tongue dorsum tissue is significantly lower than the extracted µ_s_’(λ) of the gingival tissue and labial mucosa tissue, there is no significant difference between gingival tissue and labial mucosa tissue (p = 0.60).

The reconstructed, fiber pattern-free fluorescence images for the three representative tissues are presented in Fig. 4c. The inverted and enhanced images were further processed to extract the N/Cs of the tissues. The average N/C (over all sites of the same tissue type) is 4.4 ± 0.6 (%) for gingival tissue, 4.1 ± 0.5 (%) for tongue dorsum tissue and 3.9 ± 0.8 (%) for labial mucosa tissue. Consequently, the results suggest that the incorporation of DRS measurements can provide diagnostically complementary information about the samples in question when FLI alone has difficulty in differentiating the tissues.

## Discussion

We have developed a dual-modality smartphone fiber-optic microendoscope that integrates quantitative DRS and high-resolution FLI into a portable, cost-effective device. The most challenging part of the design was to properly align the phone camera with the imaging components of both channels. Most smartphones have at least two camera modules, a rear camera and a front-facing one. While utilizing both cameras might reduce the alignment effort, the front-face camera has shown a lower imaging quality and less flexibility in terms of control. Using the front-facing camera requires many trial-and-error tests to obtain an acceptable alignment, and the performance may still be vulnerable to a small misalignment. Therefore, the rear camera was used for both FLI and DRS. However, using the cellphone camera adds some complexity to the design due to the imaging properties of the built-in camera lens kit. To maintain an accurate alignment, a ray-tracing simulation was performed to determine the best distances between the grating/eyepiece and cellphone camera so that the exit pupil of the image modules matched the entrance pupil of the cellphone camera. In addition, a sliding mechanism with a fixed moving distance was installed so the phone could easily and repeatably be switched back and forth smoothly between the FLI and DRS modules. The two channels were combined and protected using 3D printed parts that were precisely made for the design.

The preliminary *in vivo* measurement has shown that SmartME can noninvasively quantify the optical properties, hemoglobin concentrations and nuclear-cytoplasmic ratio of epithelial tissues. The ability to measure these tissue parameters at low cost may have a significant impact on epithelial cancer and precancer detection in low-resource settings. In a clinical study for cervical cancer detection using DRS conducted by Chang *et al*.^35^, a significant increase in the THC and a decrease in the mean µ_s_’(λ) were observed in high-grade neoplasia compared to the values of low-grade lesions and normal tissues. Hornung *et al*.^36^ and Georgakoudi *et al*.^37^ also observed a decreasing trend in µ_s_’(λ) (p = 0.16) in high-grade neoplasia using spectroscopy. In a review article by Thekkek *et al*.^17^, DRS was shown to have achieved sensitivities and specificities in the ranges of 83–92% and 80–90% for cervical precancer detection, respectively. High-resolution FLI also allows a differentiation of high-grade neoplastic changes from their low-grade and normal counterparts using the N/C. For example, a sensitivity of 86% and specificity of 87% were achieved using the N/C in differentiating high-grade lesions from nonneoplastic cervical tissues in 26 cervical cancer patients by Quinn *et al*.^19^. A large advantage of the SmartME system is that it combines the benefits of both FLI and DRS to provide complementary information that can be used to improve the sensitivity and specificity in differentiating different epithelial tissue types. A technique that is superior in one aspect may have limitations in other aspect so that its application can be limited. FLI allows high-resolution imaging of the tissue surface but has a limited penetration depth (less than a couple of hundred micrometers). For example, when inflammation is present on the tissue surface, using FLI alone to judge the malignancy of the lesion may be less effective. On the other hand, DRS can provide quantitative information about the biochemical properties of the tissue up to a few millimeters in depth by studying the light-tissue interactions within a specific volume of tissue, despite the fact that its spatial resolution is much lower than that of FLI. In our *in vivo* measurement, DRS provided tissue scattering and Hb concentration information that was helpful for differentiating the three different types of healthy oral tissues; such information would otherwise be difficult to obtain by using the FLI images alone since their N/Cs are not significantly different from each other.

The implementation of the SmartME system is intended to provide a cost-effective solution for precancerous screening, e.g., for cervical cancer and oral cancer, in resource-limited settings. The integration of optical imaging technologies in a smartphone platform can significantly reduce the cost, weight and size while maintaining a high performance. The total cost of the current SmartME device is less than $2500, and the major costs are the costs of the imaging fiber bundle, light sources and fluorescence filters. The cost may be further reduced by batch ordering and mass production, making the device even more affordable in LMICs. More importantly, the App developed for the SmartME has the ability to send the images collected on site to a cloud/server for diagnostic analysis. Therefore, a smartphone-based diagnosis could potentially be used as a point-of-care technology for telemedicine applications in LMICs where multiple clinical visits are not feasible and centralized laboratories do not exist.

We note that the current *in vivo* study using the SmartME has several limitations. First, because only one healthy subject was measured in the study, the conclusions may not be extended to all subjects. Many factors, including race, gender, age, diet and physical conditions, may lead to higher intersubject variations. It is also important to note that movement artifacts and probe-tissue contact pressure may also contribute to differences in the tissue optical properties from site to site as it is challenging to maintain a consistent pressure applied to the tissue by the endoscope^34,38–40^. Therefore, a device optimization, such as including a pressure sensor on the tip of the endoscope, may be necessary to further improve the reliability and consistency of the device. Finally, future clinical studies that include both normal/benign tissue and cancerous or precancerous tissue are necessary to fully verify the performance of the SmartME.

## Conclusion

We have demonstrated a fiber-optic microendoscope that integrates high-resolution fluorescence imaging and quantitative diffuse reflectance spectroscopy into a smartphone platform. The SmartME has a spatial resolution of ~3.5 µm for FLI and an accuracy comparable to that of a benchtop DRS system in measuring the absorption and scattering properties of tissue^34^. When used with the App, the device can be used to perform FLI and DRS of epithelial tissues, wirelessly transfer the data to a server for data analysis, and display the results that are sent back from the server to the SmartME within seconds. Our preliminary studies have demonstrated that the dual-modality SmartME can accurately characterize biological properties and provide complementary information about epithelial tissues. The potential of the SmartME for the early detection of neoplasia in epithelial tissues, especially in low-resource settings, will be investigated in a subsequent clinical study.

## Supplementary information

Supplemental Figure

## References

[1] Bray, F. *et al*. Global cancer statistics 2018: GLOBOCAN estimates of incidence and mortality worldwide for 36 cancers in 185 countries. *CA: a cancer journal for clinicians* (2018). 10.3322/caac.21492 30207593

[2] Modules, S. T. Cancer Registration & Surveillance M odules*. *US N ational Institutes of Health, National Cancer Institute* **1** (2010).

[3] Baba, A. I. & Câtoi, C. *Comparative oncology*. (Publishing House of the Romanian Academy Bucharest, 2007). 20806453

[4] Sung K-B (2003). Fiber optic confocal reflectance microscopy: a new real-time technique to view nuclear morphology in cervical squamous epithelium *in vivo*. Optics Express.

[5] Tan J, Quinn M, Pyman J, Delaney P, McLaren W (2009). Detection of cervical intraepithelial neoplasia *in vivo* using confocal endomicroscopy. BJOG: An International Journal of Obstetrics & Gynaecology.

[6] Fanfani F (2011). Narrow-band imaging in laparoscopic management of cervical carcinoma. Journal of minimally invasive gynecology.

[7] Fujii T (2010). Digital colposcopy for the diagnosis of cervical adenocarcinoma using a narrow band imaging system. International Journal of Gynecological Cancer.

[8] Fujimoto JG, Pitris C, Boppart SA, Brezinski ME (2000). Optical coherence tomography: an emerging technology for biomedical imaging and optical biopsy. Neoplasia.

[9] Gallwas JK (2011). Optical coherence tomography for the diagnosis of cervical intraepithelial neoplasia. Lasers in surgery and medicine.

[10] Li C (2014). Urogenital photoacoustic endoscope. Optics letters.

[11] Peng K, He L, Wang B, Xiao J (2015). Detection of cervical cancer based on photoacoustic imaging—the *in-vitro* results. Biomedical optics express.

[12] Schwarz RA (2008). Autofluorescence and diffuse reflectance spectroscopy of oral epithelial tissue using a depth-sensitive fiber-optic probe. Applied optics.

[13] Skala MC, Palmer GM, Vrotsos KM, Gendron-Fitzpatrick A, Ramanujam N (2007). Comparison of a physical model and principal component analysis for the diagnosis of epithelial neoplasias *in vivo* using diffuse reflectance spectroscopy. Optics express.

[14] Yu B, Ferris DG, Liu Y, Nagarajan VK (2014). Emerging optical techniques for detection of oral, cervical and anal cancer in low-resource settings. Austin J. Biomed. Eng.

[15] Ellis DI, Goodacre R (2006). Metabolic fingerprinting in disease diagnosis: biomedical applications of infrared and Raman spectroscopy. Analyst.

[16] Krishna CM (2006). Raman spectroscopy studies for diagnosis of cancers in human uterine cervix. Vibrational Spectroscopy.

[17] Thekkek N, Richards-Kortum R (2008). Optical imaging for cervical cancer detection: solutions for a continuing global problem. Nature Reviews Cancer.

[18] Pierce MC (2012). A pilot study of low-cost, high-resolution microendoscopy as a tool for identifying women with cervical precancer. Cancer prevention research, canprevres..

[19] Quinn MK (2012). High-resolution microendoscopy for the detection of cervical neoplasia in low-resource settings. PloS one.

[20] Breslauer DN, Maamari RN, Switz NA, Lam WA, Fletcher DA (2009). Mobile phone based clinical microscopy for global health applications. PloS one.

[21] Switz NA, D’Ambrosio MV, Fletcher DA (2014). Low-cost mobile phone microscopy with a reversed mobile phone camera lens. PloS one.

[22] Tseng D (2010). Lensfree microscopy on a cellphone. Lab on a Chip.

[23] Wu, C.-J., Wu, S.-Y., Chen, P.-C. & Lin, Y.-S. An innovative smartphone-based otorhinoendoscope and its application in mobile health and teleotolaryngology. J*ournal of medical Internet research* **16** (2014). 10.2196/jmir.2959 PMC3961810 24590187

[24] Jongsma, P. J. & Schölly, W. Device for coupling an endoscope to a videophone. (Google Patents, 2006).

[25] Gallegos D (2013). Label-free biodetection using a smartphone. Lab on a Chip.

[26] Wang, S. X. & Zhou, X. J. Spectroscopic sensor on mobile phone. (Google Patents, 2008).

[27] Smith ZJ (2011). Cell-phone-based platform for biomedical device development and education applications. PloS one.

[28] Beery, A. Introduction to mobileOCT’s multimodal imaging. Vol. 2014 (MobileOCT, 2014).

[29] Edwards P (2017). Smartphone based optical spectrometer for diffusive reflectance spectroscopic measurement of hemoglobin. Scientific reports.

[30] Hong X, Nagarajan VK, Mugler DH, Yu B (2016). Smartphone microendoscopy for high resolution fluorescence imaging. Journal of Innovative Optical Health Sciences.

[31] Muldoon TJ (2012). Noninvasive imaging of oral neoplasia with a high-resolution fiber-optic microendoscope. Head Neck.

[32] Elter, M., Rupp, S. & Winter, C. Physically motivated reconstruction of fiberscopic images. In *Pattern Recognition, 2006. ICPR 2006. 18th International Conference on*, Vol. 3 599–602 (IEEE, 2006).

[33] Palmer GM, Ramanujam N (2006). Monte Carlo-based inverse model for calculating tissue optical properties. Part I: Theory and validation on synthetic phantoms. Applied optics.

[34] Yu B, Shah A, Nagarajan VK, Ferris DG (2014). Diffuse reflectance spectroscopy of epithelial tissue with a smart fiber-optic probe. Biomedical optics express.

[35] Chang VT-C (2009). Quantitative physiology of the precancerous cervix *in vivo* through optical spectroscopy. Neoplasia.

[36] Hornung R (1999). Quantitative near-infrared spectroscopy of cervical dysplasia *in vivo*. Human Reproduction.

[37] Georgakoudi I (2002). Trimodal spectroscopy for the detection and characterization of cervical precancers *in vivo*. American journal of obstetrics and gynecology.

[38] Nath A (2004). Effect of probe pressure on cervical fluorescence spectroscopy measurements. journal of Biomedical Optics.

[39] Chen W, Liu R, Xu K, Wang RK (2005). Influence of contact state on NIR diffuse reflectance spectroscopy *in vivo*. Journal of Physics D: Applied Physics.

[40] Atencio JD (2009). Influence of probe pressure on human skin diffuse reflectance spectroscopy measurements. Optical Memory and Neural Networks.

