# Supplementary material for: A Dual-modality Smartphone Microendoscope for Quantifying the Physiological and Morphological Properties of Epithelial Tissues
Source: Sci Rep. 2019 Oct 31;9:15713. doi: 10.1038/s41598-019-52327-x (PMC6823483; doi:10.1038/s41598-019-52327-x)
Supplement: Supplementary file 1 — Supplemental Figure [file 41598_2019_52327_MOESM1_ESM.docx]

**A Dual-modality Smartphone Microendoscope for Quantifying the Physiological and Morphological Properties of Epithelial Tissues**

Xiangqian Hong, Tongtong Lu, Liam Fruzyna and Bing Yu ^*^

The supplemental figure below helps to explain the differences between the SmartME design and the previously reported designs.


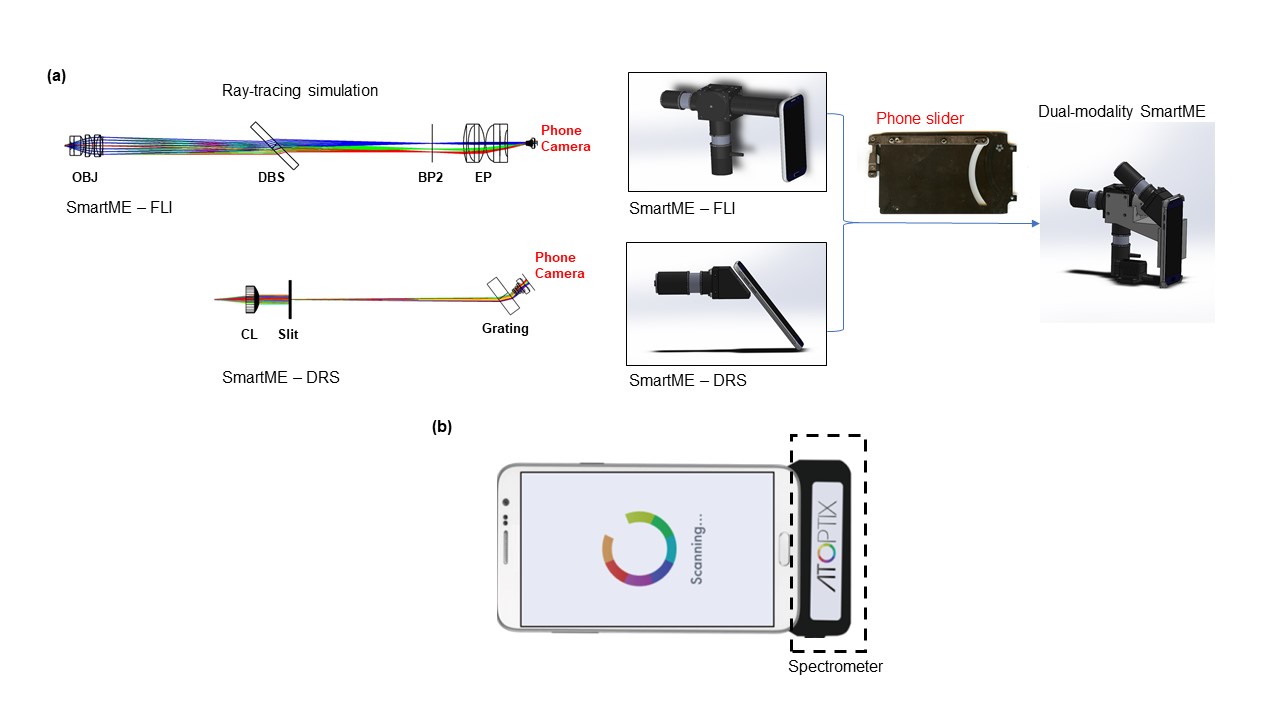


**Supplemental figure**: (a) Design process for SmartME. The SmartME uses the rear camera of a smartphone as the image sensor for both the DRS and FLI modules. Left: The distance between the phone camera and eyepiece (EP)/Grating was optimized to match the exit pupil of the FLI/DRS imaging system and the entrance pupil of the camera lens kit. Right: a phone slider was used to ensure easy switching between the two modules with minimum misalignments. (b) Design of the G-Fresnel spectrometer developed by Edwards et al. The spectrometer connects to the phone through the Micro USB port.
